# Feeding characteristics of healthy infants without reported feeding impairments throughout the first month of life

**DOI:** 10.1038/s41372-023-01760-y

**Published:** 2023-09-12

**Authors:** Katlyn Elizabeth McGrattan, Abbey E. Hammell, Morgan Elaine Turski, Kristina E. Klein, Elise Delaware, Jennie McCormick, Ellen Weikle, Erin Broderick, Sara E. Ramel, Alicia Hofelich Mohr

**Affiliations:** 1 Department of Speech Language and Hearing Science, University of Minnesota, Minneapolis, MN USA; 2 Department of Rehabilitation, Masonic Children’s Hospital, Minneapolis, MN USA; 3 Liberal Arts Technologies and Innovation Services (LATIS), University of Minnesota, Minneapolis, MN USA; 4 UC Health Department of Otolaryngology, University of Cincinnati, Cincinnati, OH USA; 5 Department of Neonatology, University of Minnesota, Minneapolis, MN USA

**Keywords:** Respiratory signs and symptoms, Nutrition disorders

## Abstract

**Objective:**

Elucidate characteristics of feeding performance in healthy infants without reported feeding problems throughout the first month of life.

**Study design:**

Feeding was monitored in 61 healthy infants by caregiver report for 48 h a week from birth to 4 weeks old. Outcomes included feeding modality, how much they consumed, how long the feed lasted, and how many coughing episodes the infant exhibited. Data were analyzed with descriptive and non-parametric statistics.

**Result:**

The majority of infants (68%) exhibited at least one problematic feeding behavior. Infants consumed 68 ml/feed over 20 min, though the milk volumes and feed durations were highly variable. Coughing occurred an average of 2 feeds per day. No significant change in coughing was observed throughout the first month of life (*p* = 0.64). Infants coughed significantly less during breast feeds than bottle feeds (*p* = 0.02).

**Conclusion:**

Healthy term infants exhibit what appear to be normal developmental imperfections in feeding performance throughout the first month of life.

## Background

Concerns regarding troublesome feeding behaviors of term infants are one of the leading sources of caregiver stress in the neonatal period [1]. A leading manifestation of these troubling behaviors is coughing during feeds, which is ranked as one of the top ten sources of postpartum stress [1]. Other manifestations include insufficient milk intake and prolonged feeding times [2, 3].

In some cases these troublesome behaviors indicate the presence of impairments in the neonate’s sucking or swallowing physiology that warrant intervention to prevent nutritional or respiratory compromise. For example, coughing during feeds is associated with pulmonary aspiration [4–7] that can cause respiratory morbidities such as wheezing, chronic cough, apnea and pneumonia [8–10]. Likewise, prolonged feeding times may be indicative of sucking deficits that limit efficient milk expression and result in hospitalization due to poor weight gain [2, 11].

In other cases, however, these behaviors cause no functional impairments [2, 5] to the infant’s well-being and appear to reflect normal developmental variations resulting from immature neurologic control [12]. Support for this theory is reflected in the relatively high prevalence of these feeding concerns among infants without functional deficits in weight gain or respiratory status [2, 8]. Survey reports indicate 10% of the aforementioned caregivers report their infants have feeding difficulties, 34% expressed concern regarding their infant’s volume of milk ingestion [2], and 25% report feeding times longer than 30 min [8]. Elucidating the specifications of what presentations delineate normal variations in performance from pathologic impairments is a critical step in the accurate diagnosis and treatment of feeding disorders. The aim of this investigation was to fill this void and elucidate normal variations in feeding performance across the first month of life using three commonly used measures of infant feeding performance: caregiver perceived feeding quality, characteristics of milk ingestion, and frequency of coughing during feeds. The hypothesis was that infants would occasionally exhibit what are clinically considered “imperfections” in their feeding performance.

## Methods

### Participants

A convenience sample of 65 healthy term infants without functional feeding deficits was recruited from the University of Minnesota Masonic Children’s Hospital postpartum unit and social media posts in this longitudinal observational investigation. Functional feeding was defined as a parent report that their infant was meeting oral nutritional needs while maintaining good respiratory health and without perceived feeding impairments. Infants were included in the investigation if they were born full term (≥37 weeks gestation) without any underlying medical conditions known to influence feeding performance and were <5 days old at the time of consent. Breast, bottle, or mixed (breast and bottle) feeding methods were all permissible forms of intake. Infants were excluded from the investigation if they had a history of feeding deficits requiring supplemental alternative nutrition (ex. nasogastric tube), poor weight gain as determined by the infant’s primary physician or feeding-induced respiratory compromise. Infants with reported tongue tie, regardless of revision, were included in the investigation as long as they were meeting the aforementioned inclusion criteria due to the variability in diagnosis methods and mixed evidence in feeding effects. Due to the uncertain feeding implications, these infants were also analyzed separately to describe potential impacts. Infants were monitored for continued adherence to eligibility criteria throughout the study period. The study was approved by the University of Minnesota institutional review board.

### Procedures

Caregivers of enrolled infants completed weekly real-time monitoring of their infant’s feeding performance 2 days a week (48 h.) for the first month of life. Monitoring was initiated at 5 days old to account for differences in feeding performance secondary to normal variations in timing of mature milk production. At each feed, caregivers recorded the feeding modality (breast, bottle or both), time the feed was initiated, number of coughing episodes that the infant exhibited during the feed, and feed end time. Coughing episodes were defined as one or more coughs that occurred in response to a stimulus. As such, if an infant was feeding, choked, and coughed 2 coughs in response, this was recorded as 1 coughing episode. If the infant returned to eating and became choked up again, this was recorded as 2 episodes. Infants fed by bottle were also monitored for volume of milk consumed and specifications of the bottle nipple used. To aid in accurate data collection and adherence, all caregivers underwent a brief training on data collection by the study team prior to initiating logging and received weekly text message reminders to start logging their infant’s feeds.

At the completion of the 48-h monitoring period caregivers received a text message with a personalized link to upload their feeding logs and provide health information regarding newly identified health conditions, their infant’s weight, and systemic health using the *Systemic Health Survey* to ensure participants continued to meet eligibility criteria. The Systemic Health Survey inquires into the presence of the following conditions: respiratory syncytial virus, diarrhea, cough/wheeze, vomiting, pneumonia, ear infection, runny nose/cold, or fever. At the end of the 1 month data collection period, the text link also prompted caregivers to provide insight on their perception of their child’s feeding performance using an early version of the *Infant and Child Feeding Questionnaire (ICFQ)* [13, 14]. The Infant and Child Feeding Questionnaire is a 12-question web-based tool designed to help caregivers discuss concerns regarding their child’s feeding performance with their pediatrician to enable earlier intervention when necessary. Since its conception it has undergone a rigorous, multi-stage psychometric testing process. The initial stage compared ICFQ responses between infants with and without feeding disorders and demonstrated the answers to four core questions, and nine negative feeding behaviors distinguished groups from one another [13]. These nine questions were used in the current investigation as this was the available version at the start of data collection. Since this time additional psychometric testing has resulting in subsequent revisions of the tool to include both an assessment and a screening version. All uploads were reviewed weekly by the study team for completeness, with discrepancies or missing information clarified by the study team contacting caregivers.

### Data analysis

Four weeks of pilot data from four participants was used to estimate weekly proportions of coughing episodes during feeds, as well as the common odds ratio of coughing episodes in week 1 compared to week 4 (OR = 4.5). A sample of 60 infants was found to be sufficient to detect this magnitude difference with 80% power with GPower software [15]. This sample size corresponds to a coefficient of variation of ~13% for mean estimates of the proportion of coughs. Given the longitudinal and intensive nature of participation, resources limited recruitment of the number of infants needed to achieve a 5% coefficient of variation for the frequency estimates (requiring ~450 infants), and to ensure sample size requirements to be met in cases of withdrawal, 65 participants were enrolled in the investigation.

Prior to data analyses health characteristics of the sample, as reported on weekly and monthly surveys, were reviewed to exclude infants who had developed the aforementioned comorbid conditions known to influence feeding performance as well as infants exhibiting functional feeding deficits during the 1-month study period. Functional feeding deficits were defined as infants whose weight was ≤2% according to the WHO weight for age growth chart or those that experienced ≥1 episode of pneumonia.

Statistical analyses were completed using R (R Core Team, 2022). Descriptive statistics were calculated for each measure with the full de-identified dataset available upon request. Ninety-five percent bootstrap, BCa confidence intervals [16] were conducted using the *np.boot* function in the *nptest* R package [17]. Inferential analyses that compared changes in infants’ feeding behavior (e.g., coughing, milk intake, etc.) across weeks were completed using the Friedman test from the R *stats* package (*friedman.test* function) [18, 19]. Collected measures of total feeding duration and total milk consumption per feed were used to calculate rate of transfer (total intake/feed duration) [20]. We limited analysis of bottle nipple flow rate and tongue tie effects to descriptive statistics due to a limited number of observations in these groups, resulting in insufficient power for any inferential analyses as well as violations of independence assumptions of the non-parametric tests. To do this, bottle nipples were categorized into previously published flow rate categories [21]: Extra Slow (<5 ml/min), Slow (5–9.99 ml/min), Medium (10–14.99), Fast (15–19.99 ml/min) and Very Fast (20–24.99 ml/min). Bottle nipples without previously published flow rate values were not included in this part of the analysis.

## Results

Sixty-one participants were consented and maintained enrollment throughout the duration of the month-long investigation and will be reported below. No participants exhibited functional feeding impairments or the emergence of comorbidities warranting exclusion during the 1-month study period. Most participants (*n* = 57, 93%) completed all 4 weeks of feeding monitoring, with the remainder (*n* = 4, 7%) missing 1 week. Eight participants (13%) had reported tongue tie, with 6 (75%) of those having undergone a frenotomy. Approximately equal proportions of infants were exclusively breastfed (41%, *n* = 25) when compared to those who received both breast and bottle feeds (59%, *n* = 36). Bottle feeds were completed using a wide array of bottles with varying nipple flow rates, with 77% completed with a bottle nipple that had previously published reference values for flow categorization. The majority of bottle feeds were completed with a slow (47%, *n* = 307) or medium flow nipple (30%, *n* = 194), and the remainder completed with a nipple that was extra slow (9%, *n* = 59) or fast (14%, *n* = 89). Table 1 provides the demographic and feeding characteristics of enrolled participants.

**Table 1:** Demographics and feeding characteristics.

Demographics	
Sex	
Male	37 (61%)
Female	24 (39%)
Race	
Black/African American	1 (2%)
Asian	3 (5%)
White	59 (97%)
Other	1 (2%)
Feeding modality	
Exclusively breast	25 (41%)
Mixed breast and bottle	36 (59%)
Bottle nipple	
Extra slow	59 (9%)
Slow	307 (47%)
Medium	194 (30%)
Fast	89 (14%)

Values shown are number of infants (%) except for bottle nipple flow rates, which depict the number of feeds (%) due to caregivers using different bottle nipples throughout the study. Percentages were rounded to the nearest whole number and may not add up to 100%. Participants could pick more than one race when asked.

All enrolled infants were in overall good health, as reported on the systemic health survey, and maintained appropriate weight gain trajectories as determined at their regular pediatrician visits throughout the study duration and weight well above the 2nd percentile for age (*M* = 56% SD = 26). When illness occurred, it was limited to mild illness including a runny nose (*n* = 3; 5%), cough/wheeze (*n* = 2; 5%), or diarrhea (*n* = 1; 2%) that resolved within 1–3 weeks. None of the infants experienced respiratory syncytial virus, vomiting, pneumonia, ear infection, or a fever.

### Parent perceived feeding quality

Perceived feeding quality, as reported by parents on the *Infant and Child Feeding Questionnaire* (*N* = 50), indicated that while all caregivers felt that their infant enjoyed being fed, 22% of caregivers often had to do something special to help their baby eat. Interestingly, 68% of caregivers indicated that their infant exhibited at least one feeding behavior that is typically considered problematic. The most frequent problematic feeding behaviors reported were falling asleep before the end of the feeding (38%), making loud breathing noises (28%), coughing (24%), and arching their body (14%). As a result of these problematic behaviors, 10% of caregivers reported having concerns about feeding their baby, though none with concerns so great that they felt they needed to seek medical attention. See Table 2 for a full listing of Infant and Child Feeding Questionnaire responses.

**Table 2:** Perceived feeding quality: Infant and Child Feeding Questionnaire (*N* = 50).

Does your baby like to be fed (Yes)?	50 (100%)
Do you think your baby eats enough (Yes)?	49 (98%)
Do you have to do anything special to help your baby eat (Yes)?	11 (22%)
Does your child often do any of the following when you feed him (Yes)?	
Refuses to eat	1 (2%)
Does not swallow	0 (0%)
Turns away from the breast, bottle, or cup	3 (6%)
Arches his body	7 (14%)
Chokes	6 (12%)
Coughs	12 (24%)
Gags	1 (2%)
Makes loud breathing noises	14 (28%)
Falls asleep before the end of feeding	19 (38%)

Values shown are number of infants (%). Percentages were rounded to the nearest whole number.

### Characteristics of milk ingestion

**Fig. 1:**
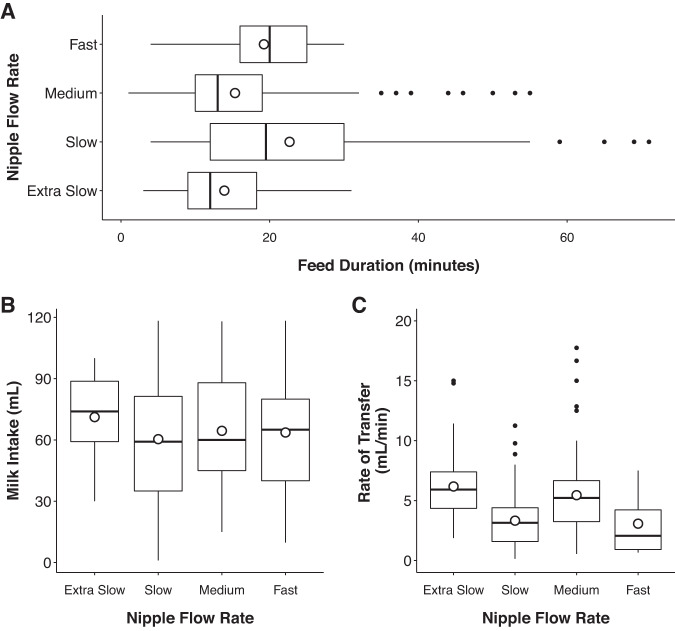
Average characteristics of milk ingestion throughout the first month of life by nipple flow. Graphs depict **A** feed duration (min), **B** milk intake (ml), and **C** rate of transfer (ml/min) by bottle nipple flow rate. The boxplots on each graph show the first, second, and third quartiles by nipple flow rate.

On average, bottle-fed infants consumed 69 ± 23 ml per feed, with each feed lasting an average of 20 ± 7 min long. This equated to an average rate of transfer of 5 ± 3 ml/min. Despite these averages there were occasions when infants would stop feeding shortly after starting (1 min) and consume minimal milk (1 ml), as well as times when they would feed over an hour (115 min). Examination of how these parameters changed throughout the first month of life revealed infants significantly increased how much they consumed with increasing age (*p* < 0.001), however they did not have significant changes in feeding duration (*p* = 0.14) or rate of transfer (*p* = 0.10). Table 3 provides a full listing of milk ingestion values throughout the first month of life. Infants with reported tongue tie performed similarly in the above outcomes to those without, consuming an average intake of 68 ml over 16 min. No clear trends were observed in the impact of bottle nipple flow rate on characteristics of milk ingestion (Fig. 1).

**Table 3:** Characteristics of weekly and monthly milk ingestion and coughing.

	1 week old	2 weeks old	3 weeks old	4 weeks old	Entire month	Friedman test
Milk ingestion						
Volume consumed (ml)	44.49 ± 16.15 (1–89)	62.70 ± 20.28 (10–163)	74.51 ± 18.33 (10–119)	80.80 ± 22.62 (10–126)	69.23 ± 22.68 (1–163)	*X*^2^ (3) = 27.33, *p* < 0.001
Feeding duration (min)	22.17 ± 8.15 (2–69)	18.79 ± 8.07 (1–105)	18.37 ± 7.19 (3–115)	18.47 ± 6.88 (1–75)	20.05 ± 7.23 (1–115)	*X*^2^ (3) = 5.56, *p* = 0.14
Rate of transfer (ml/min)	3.67 ± 1.95 (0.25–10.00)	4.85 ± 2.23 (0.87–16.67)	6.04 ± 3.06 (1.43–22.18)	5.20 ± 2.24 (1.04–17.74)	4.82 ± 3.06 (0.25–22.18)	NA
Coughing						
Feeds with at least one coughing episode	20% ± 20% (0–85%)	17% ± 16% (0–82%)	15% ± 12% (0–47%)	14% ± 13% (0–47%)	17% ± 13% (0–58%)	*X*^2^ (3) = 1.74, *p* = 0.63
Frequency of coughing in a feed if present	1.22 ± 0.37 (1–6)	1.15 ± 0.24 (1–4)	1.11 ± 0.19 (1–4)	1.16 ± 0.34 (1–4)	1.17 ± 0.23 (1–6)	*X*^2^ (3) = 2.57, *p* = 0.46

Values for milk ingestion and frequency of coughing represent mean ± SD (range). The range depicts the absolute minimum and maximum of a variable observed across all feeds within a given time period. Corresponding sample sizes and 95% BCa confidence intervals for each mean estimate above are outlined in the Supplementary Material. The Friedman test was performed on only those who had data for a given variable across all 4 weeks; this was *n* = 11 for volume consumed (bottle feed only), *n* = 32 for feeding duration, *n* = 57 for feeds with at least one coughing episode, and *n* = 44 for frequency of coughing in a feed if present. The Friedman test was not calculated for rate of transfer, as there were only four infants who had rate of transfer information across all 4 weeks.

### Frequency of coughing

**Fig. 2:**
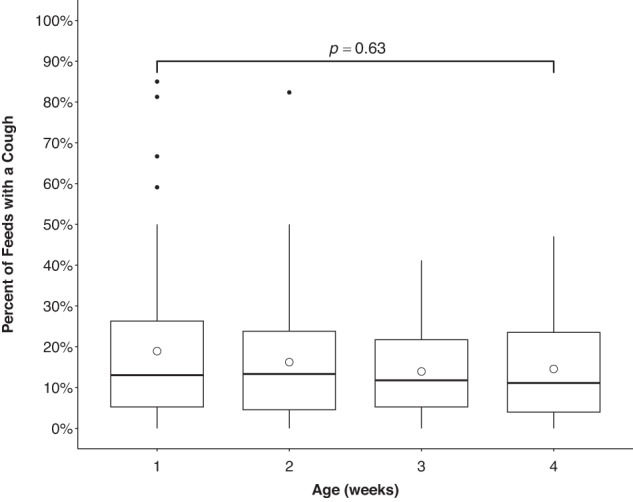
Effect of age on the percent of feeds with a cough. Boxplots show the sample’s first, second, and third quartiles. Differences in mean percent of feed with a cough among all 4 weeks were non-significant, *p* = 0.63 (Friedman test).

Almost all of the infants exhibited at least one coughing episode while feeding within the first month of data collection (*n* = 57, 93%), with the majority of infants (*n* = 38, 62%) doing so at least once during each of their weekly 48-h monitoring periods. On average, infants coughed during 17% of their feeds, equating to coughing during ~2 feeds per day. Interestingly, there was high variability in the proportion of feeds with coughing episodes across infants, with some infants not coughing at all, and one infant coughing during the majority of feeds in a given week (85%). There was no significant change in infants’ mean proportion of feeds with a coughing episode throughout the first 4 weeks of life (*p* = 0.63) (Fig. 2).

Examination of the frequency that infants coughed within individual feeds showed similar trends. Specifically, if infants coughed in a feed, it typically only happened once (*M* = 1.17, SD = 0.23) though one infant exhibited as many as six coughing episodes in a single feed. There was no significant change in the number of coughing episodes per feed throughout the first 4 weeks of life (*p* = 0.46). See Table 3 for a full listing of normal coughing values throughout the first month of life.

Significant differences in frequency of feeds with a coughing episode were found based on breast vs. bottle feeding modality (paired *t* (17) = −2.65, *p* = 0.02). Infants were significantly more likely to cough during a bottle feed (30 ± 18%) when compared to a breast feed (21 ± 15%), on average. No clear trend was observed in the impact of tongue tie or bottle nipple flow rate on the proportion of feeds with a cough. Specifically, infants with a history of tongue tie exhibited coughing episodes during 13% of feeds when compared to those without reported tongue tie who coughed during 17% of feeds. Likewise, bottle nipple impacts revealed at least one coughing episode during 3% of feeds using an extra slow flow nipple, 38% with a slow flow, 17% with a medium flow, and 14% with a fast flow.

## Discussion

In this investigation we elucidated characteristics of feeding performance among healthy breast- and bottle-fed infants throughout the first month of life using three commonly used measures of infant feeding performance. Our results demonstrated healthy infants that show no overt functional feeding impairments in weight gain or respiratory health may occasionally exhibit what are commonly considered signs of feeding impairment without suffering corresponding nutritional or cardiopulmonary sequalae associated with dysphagia. Specifically, the key findings from this investigation indicate (1) healthy infants occasionally cough during feeds; (2) infants typically finish feeding within 20 min; (3) breast feeding may offer some infants advantages in feeding quality when compared to bottle feeding.

### Healthy infants may occasionally cough during feeds

This investigation is the first to the authors’ knowledge to delineate the frequency that healthy infants without reported feeding problems cough during feeds. Results reveal it may be a normal developmental variant for healthy term infants to occasionally cough while eating. Almost all of the infants in our investigation coughed during at least one of their monitored feeds, with the average infant coughing during 2 feeds per day. Of equal importance as the average values is consideration of the full range of performance across our sample, which indicates some infants did not cough at all, while others coughed the majority of feeds. These findings relating to coughing during feeds have tremendous clinical significance, where it is common practice for clinicians evaluating infants for feeding deficits to interpret a cough during a feed as an indicator of a physiologic swallowing impairment that warrants instrumental evaluation and treatment.

Our findings suggest that an observation of coughing during feeds in itself does not always signify the presence of a swallowing impairment that warrants treatment. Instead, they support the need to evaluate the clinical significance of these coughing episodes in the context of caregiver perception of impairment, the presence of other symptoms, the infant’s respiratory health, weight gain, and the overall psychosocial components of the infant-caregiver feeding dynamic. All infants in the current investigation were in good respiratory health, following appropriate growth trajectories, and not exhibiting significant enough impairments to cause caregivers’ concern during the 1-month study period. In fact, despite nearly all infants coughing during at least one feed during the study period, the majority of caregivers did not consider those episodes as frequent or significant enough to indicate their child “coughed” [often] on the Infant and Child Feeding Questionnaire. As such, application of clinical interventions was not of clinical concern or clinically indicated. Barkmeier-Kraemer et al. and Silverman et al. found similar results in their validation work on the Infant and Child Feeding Questionnaire [13, 14]. Their work demonstrated that although children who were reported to cough during feeds were significantly more likely to have a feeding impairment than healthy controls, coughing in isolation did not identify infants with feeding impairments with accuracy. Instead, accurate identification of infants with feeding impairments required the presence of multiple indices of impairment or areas of caregiver concern [13, 14]. In light of these findings, it is critical that clinicians faced with determining the appropriate course of action after observing a cough during a feed consider the caregiver’s perception of the problem, as well as the infant’s nutritional and respiratory health, medical history, and comorbidities that place the infant at greater risk for pharyngeal or systemic health impairments if plagued by aspiration.

A key component in determining the significance of a cough during a feed is understanding its neurophysiology and developmental trajectory. The cough reflex is an airway protective response that emerges from the early laryngochemoreflex to prevent the entry of foreign materials into the lungs [22]. Previous research in human and animal models indicates this reflex can be triggered by stimulation not only to the laryngeal or tracheal region, but also the interarytenoid space and the underbelly of the epiglottis [22]. In the first weeks of life, stimulation to these regions rarely manifests as a cough, and instead commonly manifests as obstructed apnea with rapid swallows to clear the bolus [22, 23]. Within the first weeks of development, however, the maturation of the reflex causes the cough response to predominate [23]. Although it is common clinical assumption when evaluating feeding and swallowing that a cough occurs during a feed as a result of excitation of the subglottic laryngochemoreflex during an anterograde aspiration event, it is critical to keep in mind that it could also be caused by excitation of chemoreceptors in the supraglottic larynx or pharynx indicative of laryngeal penetration during an anterograde swallowing or retrograde refluxate event [24] In this case it is important to note that gastroesophageal reflux, in moderation, is an established normal manifestation of immature infant neuromuscular immaturity [25] to which the cough response is a functional healthy protective mechanism. Future investigations elucidating the source of coughing during oral feeds among healthy infants are critical in further understanding the significance of these observations.

### Infants typically finish feeding within 20 min

Our findings relating to milk ingestion also hold clinical relevance as they provide a form of calibration for clinicians to center their expectations of infant feeding performance. Clinicians commonly use 20 min as a cut-point for the duration that feeds should be in healthy infants. Feedings substantially shorter [26] or longer [27] are reported to be indices of impairment. Interestingly, there has been a paucity of investigations reporting on the actual feeding duration of healthy infants to substantiate this timepoint. Our results indicate the expectation for healthy infants to finish feeds in 20-min is valid. However, the variability in feeding duration that was observed suggests rigid expectations for feeding durations in the first month may not be a valid expectation. Though the average feeding duration was 20 min, some infants fed only for 1 min prior before falling asleep, while others fed over an hour. In fact, 38% of caregivers reported their child falling asleep before the end of the feed with many needing to stimulate the baby to finish the feed in the first weeks of life. These results raise important considerations regarding the impact of caregiver determinations on these feeding outcomes. During the first month of life caregivers experience the greatest learning curve in how to interpret their infant’s cues. As such, they may interpret their infant’s crying as an indicator that they are hungry and therefore provide a feed, when in reality the infant is crying because they are tired or agitated. As such, feedings that are brief may not reflect the infant’s abilities, but instead, reflect incorrect interpretation of the infant’s communication attempts. Likewise, the caregiver’s determination that they needed to wake their infant to finish a feed may more closely reflect the caregiver’s understandable desire to increase the child’s intake during a feeding session in effort to avoid the infant waking to feed more shortly after conclusion. Consideration of these variables should be made when interpreting caregiver’s reports and making feeding recommendations for otherwise healthy infants in the outpatient setting.

### Breast feeding may offer advantages to infant feeding quality

Our results demonstrating less coughing in infants during breast feeds when compared to bottle feeds is of great interest and potential clinical relevance. There have been few investigations comparing feeding quality between breast and bottle feeds. Hammerman et al. examined oxygen saturation in healthy term infants during and after feeds [28]. Though no difference was found between the groups during the feed, a higher proportion of infants who were bottle-fed were noted to experience oxygen desaturation below 90% SpO2 (29%) when compared to those that were breast fed (15%) (*p* < 0.05) [28]. Similar findings have been reported by Marino et al. in infants with congenital heart defects [29] as characterized by lower oxygen saturation during and after bottle feeds when compared to breast feeds [29]. Although the source for this difference is not entirely clear, one hypothesis pertains to the difference in properties of milk flow. Hydrostatic pressure within bottles makes it so milk drips regardless of sucking whenever the bottle is inverted. As such, even if an infant is attempting to take a break, the passive milk flow into their mouth make cut their break short as it initiates the swallow reflex [30, 31]. In contrast, outside of let down milk flow from the breast is restricted to times when the infant applies suction pressure. This may allow infants more time to coordinate the complex physiologic processes of swallowing needed to facilitate airway closure, better coordination breathing and swallowing, and allow for gastric emptying.

### Limitations

There are several important limitations that warrant attention in the consideration of these results. The first being these results were collected by caregivers without any video monitoring by study personnel for verification of findings. While such a methodology was necessary to enable accrual with the proposed study design, it is likely this had an impact on the provision of reporting when compared to monitoring in a lab environment. Despite the limitations in precision, this method offers the greatest clinical relevance to therapists gathering caregiver report. Another key limitation is this investigation relates to determinants on all participants in this sample being “normal” without feeding impairment. Although we took measures to ensure infants with feeding deficits would be excluded from the dataset by excluding infants with comorbidities associated with feeding impairments, those with weights that clearly place them in the category of poor weight gain, and overt impairments in respiratory health as evident by a pneumonia, this does not rule out infants with more subtle indices of functional impairment employed in the clinical setting. Likewise, as these functional impairments often do not present until after the first month, it is possible that some of the included infants would go on to encounter these problems. Our team is currently working on filling this void by following these infants longitudinally to further elucidate the characteristics of the sample identify those with feeding deficits in the future. Another key consideration in the interpretation of these findings related to respiratory health is the timing during which this study was completed. The vast majority of the participants underwent feeding and health monitoring during the peak of the COVID-19 pandemic. While COVID-19 certainly posed an exponential increase in health complications for much of the population, infants were largely unaffected by the early COVID-19 variants and in fact, had better systemic health during this time due to mandatory social distancing and mask wearing that stopped the spread of many common illnesses. This is likely reflected in our astoundingly healthy infants that rarely experienced illness as indicated on the systemic health questionnaire. Lastly, the sample size used in this investigation was used as it provided sufficient power to detect changes in feeding parameters within the first month of life, while also providing a feasible number of infants for longitudinal data collection. It is important to note that the establishment of true normative data requires substantially larger samples, often in the hundreds, that include patients with diverse demographic and social backgrounds. Future work using cohort or single time point data collection methods may enable greater accrual for more refinement in normative value establishment.

## Conclusion

Our results elucidate key attributes of healthy term infant feeding performance in the first month of life. Findings indicate slight imperfections in infant breast and bottle feeding, as characterized by some instances of coughing during feeds and occasional feeds substantially shorter or longer than the 20-min threshold are likely a normal variant of performance. These manifestations likely reflect the natural imperfections in performance that occur when an immature neuromuscular system attempts to complete a physiologically demanding task. Breast feeding may provide benefits to the infant’s immature neuromuscular system’s ability to tolerate continuous milk flows offered by bottles. Attention to the infant’s ability to meet functional feeding needs, as characterized by full oral intake while maintaining cardiopulmonary stability and enjoyable infant and caregiver feeding interactions, is of critical importance in determining the need to implement dysphagia interventions.

### Supplementary information

Supplementary materials

## Data Availability

The de-identified data that support the findings of this study are available upon request.
