# Supplementary material for: Feeding characteristics of healthy infants without reported feeding impairments throughout the first month of life
Source: J Perinatol. 2023 Sep 12;44(1):71–7. doi: 10.1038/s41372-023-01760-y (PMC10783545; doi:10.1038/s41372-023-01760-y)
Supplement: Supplementary file 1 — Supplementary materials [file 41372_2023_1760_MOESM1_ESM.docx]

| **Supplemental Table 1. Milk Ingestion** |  |  |  |  |
| --- | --- | --- | --- | --- |
|  | *M* | *SD* | *95% CI* |  |
| *Average volume consumed per feed (mL)* |  |  |  |  |
| Full Month (*n* = 34) | 69.23 | 22.68 | [61.85, 76.99] |  |
| Week 1 (*n* = 17) | 44.49 | 16.15 | [36.86, 51.62] |  |
| Week 2 (*n* = 20) | 62.7 | 20.28 | [55.81, 74.02] |  |
| Week 3 (*n* = 25) | 74.51 | 18.33 | [67.46, 81.47] |  |
| Week 4 (*n* = 24) | 80.8 | 22.62 | [71.75, 89.38] |  |
| *Average feeding duration (minutes)* |  |  |  |  |
| Full Month (*n* = 46) | 20.05 | 7.23 | [18.10, 22.15] |  |
| Week 1 (*n* = 39) | 22.17 | 8.15 | [19.79, 24.80] |  |
| Week 2 (*n* = 41) | 18.79 | 8.07 | [16.59, 21.60] |  |
| Week 3 (*n* = 43) | 18.37 | 7.19 | [16.50, 20.79] |  |
| Week 4 (*n* = 39) | 18.47 | 6.88 | [16.45, 20.79] |  |
| *Average rate of transfer (mL/min)* |  |  |  |  |
| Full Month (*n* = 22) | 4.82 | 3.06 | [3.85, 6.49] |  |
| Week 1 (*n* = 10) | 3.67 | 1.95 | [2.52, 4.76] |  |
| Week 2 (*n* = 12) | 4.85 | 2.23 | [3.80, 6.24] |  |
| Week 3 (*n* = 15) | 6.04 | 3.06 | [4.97, 8.26] |  |
| Week 4 (*n* = 15) | 5.2 | 2.24 | [4.27, 6.48] |  |
| *Note.* n refers to the number of infants in the analysis for the given week (or entire month). 95% CIs were calculated using the BCa method in the np.boot() function in the nptest package in R. | | | |  |
|  |  |  |  |  |
|  |  |  |  |  |
|  |  |  |  |  |
|  |  |  |  |  |
|  |  |  |  |  |

| **Supplemental Table 2. Coughing** |  |  |  |  |
| --- | --- | --- | --- | --- |
|  | *M* | *SD* | *95% CI* |  |
| *Proportion of feeds with at least 1 coughing episode* |  |  |  |  |
| Full Month (*n* = 61) | 0.165 | 0.127 | [.137, .200] |  |
| Week 1 (*n* = 59) | 0.200 | 0.205 | [.156, .261] |  |
| Week 2 (*n* = 61) | 0.168 | 0.163 | [.134, .217] |  |
| Week 3 (*n* = 61) | 0.146 | 0.116 | [.119, .177] |  |
| Week 4 (*n* = 59) | 0.142 | 0.130 | [.111, .177] |  |
| *Frequency of coughing in a feed if cough present* |  |  |  |  |
| Full Month (*n* = 57) | 1.17 | 0.23 | [1.12, 1.24] |  |
| Week 1 (*n* = 48) | 1.22 | 0.37 | [1.14, 1.35] |  |
| Week 2 (*n* = 51) | 1.15 | 0.24 | [1.09, 1.23] |  |
| Week 3 (*n* = 53) | 1.11 | 0.19 | [1.07, 1.17] |  |
| Week 4 (*n* = 44) | 1.16 | 0.34 | [1.09, 1.32] |  |
| *Note*. *n* refers to the number of infants in the analysis for the given week (or entire month). 95% CIs were calculated using the BCa method in the *np.boot()* function in the nptest package in R. | | | |  |
|  |  |  |  |  |
|  |  |  |  |  |
|  |  |  |  |  |
|  |  |  |  |  |
